# Time consciousness: the missing link in theories of consciousness

**DOI:** 10.1093/nc/niab011

**Published:** 2021-04-12

**Authors:** Lachlan Kent, Marc Wittmann

**Affiliations:** Centre for Youth Mental Health, The University of Melbourne, 35 Poplar Rd, Parkville, Victoria 3052, Australia; Orygen, 35 Poplar Rd, Parkville, Victoria 3052, Australia; Institute for Frontier Areas of Psychology and Mental Health, Wilhelmstraße 3a, 79098 Freiburg i.Br., Germany

**Keywords:** time consciousness, time passage, experienced present, consciousness theories

## Abstract

There are plenty of issues to be solved in order for researchers to agree on a neural model of consciousness. Here we emphasize an often under-represented aspect in the debate: time consciousness. Consciousness and the present moment both extend in time. Experience flows through a succession of moments and progresses from future predictions, to present experiences, to past memories. However, a brief review finds that many dominant theories of consciousness only refer to brief, static, and discrete “functional moments” of time. Very few refer to more extended, dynamic, and continuous time, which is associated with conscious experience (cf. the “experienced moment”). This confusion between short and discrete versus long and continuous is, we argue, one of the core issues in theories of consciousness. Given the lack of work dedicated to time consciousness, its study could test novel predictions of rival theories of consciousness. It may be that different theories of consciousness are compatible/complementary if the different aspects of time are taken into account. Or, if it turns out that no existing theory can fully accommodate time consciousness, then perhaps it has something new to add. Regardless of outcome, the crucial step is to make subjective time a central object of study.

## Introduction

The recent ascent of theories of consciousness has undoubtedly raised the stakes regarding fundamental aspects of experience and reality. Within this high-stakes environment, where competing or even adversarial perspectives are vying for ascendancy, the fundamentals in questions have to be clear, concise, and consistent. One prime example is time or, in its context-specific form, time consciousness. While there is a prevailing consensus in the field that consciousness is extended in time (Northoff and Lamme 2020), in our opinion as dedicated time researchers, it is not yet extended enough. Decades of timing research supports a “minimally sufficient” duration for time consciousness somewhere in the seconds’ range (Fraisse 1984; Pöppel 1989, 1997; Varela 1999; Wittmann 2011; Kent 2019), but most theories and methodologies in consciousness science only focus on the hundreds-of-milliseconds’ range (Northoff and Lamme 2020). As such, we claim that current theories do not adequately address time as a fundamental aspect of conscious experience. The discrete “timing” of brief neural, perceptual, and behavioral functioning cannot hope to explain time consciousness when, from a prevailing phenomenological viewpoint, it is neither discrete nor brief (Wittmann 2016; Dorato and Wittmann 2020).

One possible misconception at the root of this problem is that time consciousness is synonymous with the *timing* of behavior, perception, and other stimulus-based responses or event-based experiences. Timing and other nonconscious aspects of time perception should not be confused or conflated with time consciousness itself, which can be defined as the conscious experience *of time*, as opposed to events (e.g. a perceptual stimulus) that merely happen *at specific times* (Kent 2019). This distinction is about the general feeling we have of time passing from the future to the past (sometimes as the impression of time passing slowly or fast), on the one hand, and the sensorimotor timing of behavior in relation to the duration of specific events, on the other hand. The former is concerned with the phenomenal impression of subjective passage of time, the latter with the comparison between subjective duration and objective clock time, i.e. the accuracy of estimating the duration of an event.

Taking a neuroscientific approach as well, there are in fact three separable but interdependent “times” at play in the study of time consciousness—neural temporal dynamics, functional timing of perception/thought/behavior, etc., and the phenomenal experience of time—all of which must be addressed in any general neuroscientific theory of consciousness. The goal must be to arrive at a theory that simultaneously explains how underlying neural dynamics *in time* generate a conscious experience *of time*, how the experience *of time* affects how people perceive, think or act *at specific times*, and how those perceptions, thoughts, and actions feedback to shape (or a complementary to) the underlying neural (temporal) dynamics and conscious experience. Neural, functional, and phenomenal aspects of time need to be triangulated in order to understand the key features of time consciousness, two of which we focus on below—namely, extension/duration and continuity/flow.

## Time Consciousness Is Grounded in Phenomenology

William James’ (1890) “stream of consciousness” and Edmund Husserl’s (1928/1991) “inner time consciousness” attempt to explicate phenomenologically that all experience happens within an extended present, a unified temporal and spatial whole of experience, within which the unfolding of events, time passage, happens. The experienced present is extended as it carries an event’s history and possible future within the implicit temporal structure of consciousness (Lloyd 2012). Edmund Husserl (1928/1991, 32) writes about the duration of the temporal field, “which is manifestly limited, precisely as in perception’s case. Indeed, on the whole, one might dare to assert that the temporal field always has the same extensions.” As empirical researchers we may ask what the extension of this field of consciousness is, e.g. in units of clock time. Some contemporary theorists deny that consciousness extends over a relatively fixed “specious present” on either empirical (White 2017) or philosophical (Arstila 2018) grounds. It is by no means a settled question, but the early phenomenological and experimental studies of Husserl (1928/1991) and James (1890) are supported by contemporary theory and research (Zahavi 2005; Lloyd 2012; Berkovich-Ohana and Glicksohn 2014; Northoff 2016). If consciousness is extended in time and possesses the experiential quality of time passage, we have to probe neural and functional theories of consciousness concerning their answers to this issue.

Building on Husserl’s and others’ phenomenological conception, three temporal aspects of conscious experience can be discerned, two of which we will address in our article, namely the extended present, time passage or flow, and one which we will not address in detail, namely the tripartite structure concerning past, present, and future. These aspects have been related to neuronal and psychological properties in empirical science. First, an experienced present has been operationalized to account for the feeling of an extended now and sensorimotor integration with perception and behavior (Pöppel 1997; Wittmann 2011; Tschacher *et al.* 2013; Kent 2019). As Edelman (1991, 92) argues, the constant flux and variation of incoming sensory signals necessitate a temporal organization, a “cohesion in a given period of time” potentially afforded through reentrant processing, for primary consciousness to arise. Second, experimental paradigms have been set up to account for the feeling of time passage in healthy individuals and the corresponding experiential and functional breakdown in patients with schizophrenia (Lalanne *et al.* 2010; Martin *et al.* 2014). Unconscious processes may enable us to follow information over time with high temporal accuracy and at the same time contribute to our feeling of time passage (Elliott and Giersch 2016). Third, human experience can be categorized into the temporal domains of past, present, and future which on a sensorimotor-processing level correspond to the cognitive functions of working memory, interference control, and preparatory set, respectively (Fuster 2003), and arguably to Husserl’s phenomenological notion of *retention*, *impression*, and *protention* (Vogeley and Kupke 2006).

## Duration: Time Consciousness Is Extended

The orthodox view in the cognitive neurosciences is that time consciousness extends over a duration between a few hundred milliseconds and a few seconds over what is otherwise called the “experienced moment” or “subjective present” (Pöppel 1997; Wittmann 2011; Kent 2019). Experimental findings discussed in the above reviews indicate that the perception of external events is automatically segmented into units with a duration of a few seconds such as when listening to sequences of beats, when viewing ambiguous figures, or when viewing distorted naturalistic visual sequences (Nakajima *et al.* 1980; Fairhall *et al.* 2014; Wang et al. 2016; Kornmeier *et al.* 2019). These special stimulations are supposed to be limit cases of general processing mechanisms which temporally structure all of our perception and action (Pöppel 1997). Sensorimotor synchronization to external events is effortless and most accurate when one has to time movements to regular events with intervals not exceeding three seconds (Mates *et al.* 1994; McAuley *et al.* 2006). There is a debate concerning the question whether several independent mechanisms with variable duration underlie all these different experimental paradigms (White 2017), they nevertheless point to a similar time range and could be related to what Husserl termed the “temporal field” of an experienced present. Humphrey (1992) similarly advocates the idea of present moment-ness. According to him, repeated feedback processes of neural sensory-motor loops induce perceptual content, which is extended over time. Consciousness thereafter is “thick,” i.e. an extended perception of what is out there.

Empirical evidence stemming from behavior analysis and using brain imaging technology has identified neural integration processes on different time scales. These temporal integration processes define how long a given event proactively influences moment-to-moment neural activity stimulated by continuous visual and acoustic input (Hasson *et al.* 2008; Lerner *et al.* 2011). However, one has to be careful in identifying subjective experience with neural integration processes, as the latter might represent functional properties of the brain unrelated to the experienced present (Lockwood 2005). This principle applies at all timescales of human experience, too. We have (conscious) experiences that span hours, days, and even decades, but this should not be identified solely with temporal integration processes given that there are memory and other higher-order processes involved (Kent *et al.* 2019). This is the decisive goal for forthcoming research: if possible, to identify the exact neural correlates of present moment experience. Importantly, the neural mechanisms underlying consciousness are not stimulus bound but act in a top-down anticipatory fashion. “When in a conscious state, experience will unfold regardless of specific stimulation, because stimulation is no strict requirement for conscious experience given the predictive inferential machinery” (Fekete *et al.* 2018, 7).

Notwithstanding recent neuro-phenomenal work in time consciousness that seeks to explain the “width of present” in terms of the continuous, singular nature of conscious experience (Northoff 2016), we argue in this article that all other theories of consciousness are generally confined to the functional moment because they are derived from methods that restrict activity within that short timescale range. In their review article on eight different neural theories of consciousness, Northoff and Lamme (2020) show that only Northoff’s (2016, Northoff and Huang 2017) Temporo-spatial theory of consciousness (TTC) explicitly incorporates temporal integration mechanisms on longer time scales of seconds to minutes duration. The other theories only address short temporal integration on the millisecond level, i.e. between 100 and 300 ms. We argue that this confinement leads to intractable problems within and between theories of consciousness, citing the recent controversy surrounding the “unfolding argument” as a prime example (Kleiner 2019; Doerig *et al.* 2019b; Ganesh 2020; Negro 2020; Tsuchiya *et al.* 2020).

## Flow: Time Consciousness Is Continuous

Whatever the actual extent of the experienced moment and its underlying neural mechanisms, the original and principal distinction captured in the concept of time consciousness is between punctate, point-like present *moments* (plural) on the one hand, and the integration of those moments into an extended, field-like present *moment* (singular) on the other hand (Montemayor and Wittmann 2014). Perception and action evolve as discontinuous processing of discrete momentary units (Pöppel *et al.* 1990; Dehaene 1993; VanRullen and Koch 2003). These moments are characterized by the idea of co-temporality, events within such a time unit have no before–after relation (Ruhnau 1995). Speech recognition, e.g. is enabled through the 3–6 Hz segmentation of the continuous speech stream into temporal units for perceptual and linguistic analyses (Teng *et al.* 2019). Neural microstates as recorded by electroencephalogram of around 125 ms duration have, e.g. been discussed as potential critical time windows, as “atoms of thought” which functionally integrate neural events across the cortex (Lehmann *et al.* 1998; Milz *et al.* 2016). These nonconscious, discrete “functional moments” are ascribed by their dynamic range over a few hundred milliseconds, as opposed to the latter conscious and continuous “experienced moment” that ranges over a few seconds (Stern 1897; Fraisse 1984; Pöppel 1997; Varela 1999).

In short, continuity entails temporal *flow* between discrete temporal units. Discrete time refers only to a *minimally* extended present that can be thought of as a basic temporal “unit” (cf. a functional moment). Discreteness does not apply to a *maximally* extended present that requires passage or flow *between* discrete time units. Such maximal extension is continuous by definition, in that it could not possibly be otherwise because it entails the co-consciousness of percepts that are not simultaneous (cf. co-temporal, as above). That is, extended experiences exhibit temporally ordered structure but are nevertheless perceived as a unified whole (Kiverstein and Arstila 2013; Dorato and Wittmann 2020). An example often cited is how people hear the musical phrase “Hey Jude” within a separable but unified perceptual whole that holds both *discrete* units, “Hey” and “Jude,” within the same *continuous* experience of the full “Hey Jude” phrase (Lloyd 2012).

Any theory of consciousness that aims to include time consciousness must therefore explain how the brain achieves this feat at a neural level. Our analysis below suggests that, with only one or two exceptions (Northoff and Lamme 2020), many of the leading candidate theories cannot explain continuity or flow because they are methodologically (and thus theoretically) constrained to short, discrete, nonconscious functional moments.

## Consciousness, Time, and Self: The Importance of No-Stimulus Paradigms

Theories of consciousness have been constrained methodologically. Prevailing research methods almost exclusively use stimulus-based paradigms when searching for the neural correlates of consciousness (NCCs; Koch 2004) but time itself is not consistently controlled in the vast majority of experiments and not in-keeping with current notions regarding the actual extension (cf. duration) of conscious experience. Temporal processing of stimuli can be probed in specific tests (i.e. gap detection, temporal order judgements, stimulus duration perception), typically in the order of milliseconds to a few seconds, but “no-stimulus” paradigms can be devised to judge empty intervals of much longer duration (Thönes and Stocker 2019). Such experimental setups, e.g. are those where one has to wait through a period of time for something to happen or to end. Temporal judgments and prediction in these studies can range from the sub-second (Martin *et al.* 2017), to the multiple second (Röhricht *et al.* 2018), and several minutes’ range (Pfeifer and Wittmann 2020).

It is therefore possible to close the gap between theories of consciousness and time consciousness by using “no-stimulus” paradigms which strike at the common heart of time, self, and conscious experience. “No-stimulus” paradigms are central to the study of time consciousness because research has demonstrated that time perception, especially of longer duration, is intimately linked to the perception of self as unfolding over time, such that an increased awareness of self corresponds to an increased awareness of time, and vice versa (Wittmann 2015). Whether this is tied to the basic, immediate, embodied sense of self-hood akin to interoceptive cues over short timescales from a few-hundred milliseconds up to several seconds (Craig 2009a, b; van Wassenhove et al. 2011), or the narrative, autobiographical sense of self-hood spanning longer timescales of months, years, and decades (Conway *et al.* 2004; Bird and Reese 2006), both types of self-awareness are central, if not necessary, to any definition of consciousness as “what it is like to be something” (Nagel 1974). A disturbed sense of self is typically associated with a disturbed sense of time (Fuchs 2013; Martin *et al.* 2014).

## Summary of Time Consciousness

Time is a complex topic, especially for the uninitiated who specialize in general theories of consciousness. Before discussing time in theories of consciousness, it may help to review the key aspects of time consciousness:

- Time consciousness should not be: (i) confused with timing of cognitive functions; or (ii) identified with all timescales of temporal integration.
- Time consciousness extends over multiple seconds, not just a few-hundred milliseconds.
- Time consciousness is not discrete or point-like (i.e. it does not “happen” at a particular time) but rather field-like (i.e. it contains multiple points that happen at different times but are nevertheless experienced together).
- Short and discrete “functional moments” that are *nonconscious* are integrated into longer and continuous “experienced moments” that are conscious.
- This continuous integration results in the phenomenal sense of temporal flow in conscious experience.
- Neuroscientific approaches to consciousness do not apply these established principles of time consciousness consistently and so theories of consciousness and time consciousness are potentially incommensurate.

## Short, Discrete, and Static: The Current Status of Time in Theories of Consciousness

After a proliferation of alternative theories of consciousness in recent decades, the current trend is toward parsimony through synthesis (Waade et al. 2020), standardization (Graziano *et al.* 2020), or direct competition (Reardon 2019). In this vein, Northoff and Lamme (2020) reviewed the convergence between eight theories of consciousness (i.e. integrated information theory [IIT], global neuronal workspace theory [GNWT], predictive coding theory [PCT], recurrent processing theory [RPT], embodied theory [ET], synchrony theory [ST], higher-order thought theory [HOT], and temporospatial theory [TTC]) and included details about the “timing of consciousness”. While TTC, ET, PCT, and HOT were less constrained, their review found that major theories such as IIT, GNWT, RPT, and ST were concerned only with narrow timescales between approximately 100 and 300 ms. They noted that GNWT deals with comparatively “late” processing that is beyond 300 ms but, as discussed below, in reality, this late processing does not extend sufficiently far beyond 300 ms into the seconds’ range.

Their analysis further hints at the possibility that GNWT, RPT, and ST may be methodologically constrained by a shared reliance on stimulus-based paradigms such as masking, binocular rivalry, change/inattentional blindness, attentional blink, and so on (Northoff and Lamme 2020). The reason why IIT is limited to particular timescales under 100 ms is less clear but, regardless of the reasons why, the fact remains that several of the most prominent theories of consciousness do not currently allow enough time for extended and continuous time consciousness to occur. On the plus side, some theories seem compatible with time consciousness (i.e. ET, PCT, and HOT) and TTC (Northoff and Huang 2017) is firmly dedicated to more expansive timescales of consciousness, including infra-slow (0.0001–0.1 Hz) and slow (0.1–1 Hz) neural oscillations. But there is still scope for improvement, especially for prominent theories like IIT and GNWT. These two theories will now be explored in detail in relation to controversies surrounding the “unfolding argument” and discrete versus continuous perception (Doerig *et al.* 2019a,b; Drissi-Daoudi *et al.* 2019).

## Time Consciousness in IIT and GNWT

IIT posits a mathematical framework for the quality and quantity of consciousness from a phenomenological starting point that details properties of conscious experience (cf. intrinsicality, composition, information, integration, and exclusion; Tononi *et al.* 2016). The last of these properties, exclusion (cf. experience is definite in its content and spatiotemporal grain), grapples with phenomenal aspects of time consciousness in terms of duration and flow. In recent formulations of the theory, Tononi and Koch (2015, 6) state clearly that “experience flows at a particular speed—each experience encompassing a hundred milliseconds or so—but I am not having experience that encompasses just a few milliseconds or instead minutes or hours.” This particular estimate of a hundred milliseconds is shorter than most estimates from time consciousness research (Kent 2019; Dorato and Wittmann 2020) and so we would question whether time as operationalized in IIT is indeed a phenomenologically extended present (cf. experienced moment). Tononi *et al.* (2016) later expanded this estimate to state that the “duration of the instant of consciousness is also definite, ranging from a few tens of milliseconds to a few hundred milliseconds, rather than lasting a few microseconds or a few minutes” (p. 452), but even this timescale remains within only the lower proposed ranges of the experienced moment (Kent 2019) and is still framed as a discrete, duration-less “instant” of time. IIT falls short of the standard operational definition of time consciousness ranging between durations of a few hundred milliseconds up to a few seconds.

Interestingly, this constraint was not as evident in earlier formulations of IIT. Tononi (2004) was fully aware of the need to specify “the time requirements on neural interactions that support consciousness” if, according to the theory, “each particular conscious experience is specified by the value, *at any given time*, of the variables mediating informational interactions among the elements of a complex” (cf. the value of Φ or “phi”, p. 1, emphasis added). Tononi (2004, 3) acknowledged the difference in duration between functional moments and the experienced moment, and also the issue of discrete versus continuous experience:

Studies of how a percept is progressively specified and stabilized – a process called microgenesis – indicate that it takes up to 100–200 milliseconds to develop a fully formed sensory experience, and that the surfacing of a conscious thought may take even longer… Other evidence indicates that a single conscious moment does not extend beyond 2–3 seconds…While it is arguable whether conscious experience unfolds more akin to a series of discrete snapshots or to a continuous flow, its time scale is certainly comprised between these lower and upper limits. Thus, a phenomenological analysis indicates that consciousness has to do with the ability to integrate a large amount of information, and that such integration occurs at a characteristic spatio-temporal scale.

The question, then, is why there has been a shift in emphasis between earlier and later versions of IIT. Consistent with Tononi *et al.* (2016), the review by Northoff and Lamme (2020) concluded that IIT concerned only early processing after stimulus onset (100–300 ms). The key is “after stimulus onset,” meaning that the methodological application of IIT has constrained the theory due to the reliance on stimulus-based paradigms. It is not within the scope of this article to explore the temporal characteristics of these methodologies in detail, and so we take it on face value (i.e. the authors’ words and reviewers’ findings) that IIT deals almost exclusively with short, discrete functional moments, despite Tononi (2004) acknowledging that a “single conscious moment” can extend up to a few seconds. As Fekete *et al.* (2016) point out, IIT needs to show how the property of exclusion is dynamic and continuous over a typical spatiotemporal grain of experience across spatial and temporal scales.

The impact of this issue for IIT may extend beyond mere methodology, too. In order to calculate a value of Φ and the quantity of consciousness “at any given time,” IIT relies on a *synchronic*, summative approach that assumes a point-like value at time *t*. This can then be compared with another time *t'* and a conclusion can be made about the amount of consciousness based on comparison between the two timepoints. This is a problem for time consciousness that is field-like as opposed to point-like, because a temporal field contains multiple points, and so multiple Φ’s, meaning that the value of Φ “at any given time” is not a single value that can be compared veridically. Using an alternative *non-synchronic* (i.e. diachronic), nonsummative approach to calculating Φ requires a notion of dynamic change or spread of values. In TTC, Northoff and Huang (2017) use a measure of intrinsic temporal autocorrelation of neural activity spanning across multiple timescales from milliseconds, to seconds, to minutes to describe a “repertoire” of “scale-free dynamics” that is not bound to the onset of a paradigmatic stimulus. This approach has already been applied to explain the breakdown of consciousness in general anesthesia (Zhang et al. 2018), and isolated studies have examined resting-state functional temporal dynamics during sleep in relation to IIT and GNWT (Tagliazucchi *et al.* 2013). Perhaps IIT (and GNWT and other theories of consciousness) could better utilize this kind of temporal scale-free approach to calculating Φ and, in doing so, return to the expanded timescales of conscious experience cited in early formulations of the theory (Tononi 2004). The important feature is that dynamics need to be construed over a continuous range of hierarchically nested timescales with a particular spatiotemporal grain (Fekete *et al.* 2016; Kent *et al.* 2019).

Such an approach could also help to clarify or resolve controversies such as the recent “unfolding argument” proposed by Doerig *et al.* (2019b), which criticized IIT and other causal structure theories of consciousness on the grounds that recurrent feedback neural networks could be “unfolded” (i.e. either partly or wholly replaced) by nonrecursive feedforward neural networks, undermining the central claim of IIT that recurrent networks are a necessary feature of consciousness. It is important to note that the authors use “unfolding” in a spatial sense, rather than temporal. A system is unfolded into *n *+* m* layers if it functions the same as a recurrent system with only *n* layers. So the unfolding only adds spatial layers to the configuration. Temporally, this unfolding still happens within discrete moments of time such that the system receives an input (I) at time *t* and then later gives an output (O) at time *t'*. Both recurrent and feedforward systems can do this (for every I and O), so the difference is in *how* they do it (cf. internal spatial organization), not *when*.

While not a major theme within the many responses to the unfolding argument to-date (Kleiner 2019; Ganesh 2020; Negro 2020; Tsuchiya *et al.* 2020), we believe that discrete time (i.e. time *t* and *t'*) is at the core of this controversy. Doerig *et al.*, the authors of the unfolding argument, are also proponents of discrete over continuous perception (Doerig *et al.* 2019a; Drissi-Daoudi *et al.* 2019; Herzog *et al.* 2020), a position which seems to align with their views on consciousness. They recently proposed hard criteria for theories of consciousness, and evaluated existing theories against these criteria, without addressing the distinction between discrete perception versus continuous experience (cf. perception and action, as above) (Doerig *et al.* 2020). The problem is not that the issue is unresolved, the mechanisms behind continuous perception remain opaque, but that the authors maintain both positions (cf. unfolding in space and discreteness in time) from a common philosophical standpoint that explicitly denies phenomenological grounding:

(T)emporal features, such as motion, are not consciously perceived while they occur. They are not even perceived over an extended period of time, but are encoded as any other feature, such as colour or shape, by a *static* label. For example, motion is not represented by a signal that moves in time but by the output of a motion detector. (Doerig *et al.* 2019a, 2; emphasis in original)

Time in their view is static, discrete, and restricted to short timescales (depending perhaps upon unconscious, quasidynamic processes which are integrated into discrete moments with duration up to 450 ms; Herzog *et al.* 2016) but, as above, this is at odds with time consciousness or would necessitate an additional mechanisms to account for functional and phenomenal temporal integration over several seconds. Like IIT, discrete perception here is equivalent to functional moments, which are short, discrete, preconscious building blocks that define simultaneity. Doerig *et al.* (2019a) make their opposition to phenomenal continuity explicit by denying that *any* experience of motion is extended in time:

The question about the time course of perception directly relates to the question of qualia. As mentioned, motion detection does not need to be coded by a dynamically changing representation. What about motion *experiences*? In our model, the experience of motion does not extend in time, it only *seems* to. (Doerig *et al.* 2019a, 3; emphasis in original)

From a phenomenological standpoint of time consciousness, the very fact that motion “seems to” extend in time is the very thing that needs to be explained (subjective experience). Ultimately, the authors concede their position is based on philosophical underpinnings that approximate the illusionist position of Dennett (2016) and Frankish (2016).

Proponents of IIT could counter these claims by abandoning the synchronic calculation of Φ in favor of a more dynamic, diachronic definition of integrated information. This empirical shift could bridge the ideological divide and seeming philosophical impasse between qualia realism and illusionism, as well as those who deny or affirm a phenomenology-first approach. If IIT predictions do not accord with the integration of information and conscious states over timescales of the experienced present, which is extended, then those predictions and some of the theory could be considered falsified. In response to the unfolding argument, Negro (2020, 7) proposes that IIT would be at least partially falsified if “the informational structure did not change at the same temporal scale of the stimulus and the phenomenal experience, or, even worse, if it did not change at all.” This is because causal structure theories like IIT require strict isomorphism between phenomenal and physical structures (Tsuchiya *et al.* 2020). Taking the stimulus out of the picture, the phenomenology of time consciousness also requires that the informational structure be extended and made continuous in order to achieve that strict isomorphism.

The GNWT proposes that a high-level unified space, where information is shared and broadcast back to lower processing levels, providing global access to a range of nonconscious perceptual, memory, attentional, motor, and evaluative aspects of conscious experience (Dehaene and Changeux 2011). The authors characterize the top-down integrative process as “slow and late” (Dehaene and Changeux 2011, 215), but not slow or late enough to be considered within the timescale of the experienced moment:

A strong statement of the proposed theoretical synthesis is that early bottom-up sensory events, prior to global ignition (< 200–300 ms), contribute solely to nonconscious percept construction and do not systematically distinguish consciously seen from unseen stimuli […] Whether it takes 200 ms, 300 ms, or even more, the slow and integrative nature of conscious perception is confirmed behaviorally by observations such as the ‘‘rabbit illusion’’ and its variants […], where the way in which a stimulus is ultimately perceived is influenced by poststimulus events arising several hundreds of milliseconds after the original stimulus. Psychophysical paradigms that rely on quickly alternating stimuli confirm that conscious perception integrates over ∼100 ms or more, while nonconscious perception is comparatively much faster […]. Thus, whether externally induced or internally generated, the ‘‘stream of consciousness’’ may consist in a series of slow, global, and transiently stable cortical states.” (Dehaene and Changeux 2011, 215)

Besides being brief, the visual masking, attentional blink, inattentional blindness and other paradigms used to test GNWT focus primarily on stimulus-related neural processing (P300 or P3b) associated with the “global ignition” of a conscious percept (Northoff and Lamme 2020), which imposes only a discrete functional “cut-off” point. While the authors of GNWT refer to the “stream of consciousness” as a series of cortical states, the fact that it is only a series confirms that global ignition refers to discrete (functional) moments and does not propose a basis for the continuity of time consciousness. More recent experimental work in the GNWT tradition continues this trend by locating discrete all-or-none temporal sampling of visual information at around the 350-ms timescale range (Marti and Dehaene 2017). Again, this emphasis on discreteness is probably related to the short timescale range of the durations involved that span the upper limit of functional moments (Kent 2019).

It is clear that both IIT and GNWT are not currently amenable to the notion of an extended or continuous time consciousness as outlined above. The functional moment as captured by these two theories is too instantaneous, short, and discrete to capture the phenomenology of time (Dorato and Wittmann 2020). That is a strong claim but to take phenomenology seriously in consciousness research means grounding experimental findings in first-person accounts of their own (cf. our own) experience (Negro 2020). Do you/I/we experience discrete or continuous temporal flow? Is the stream of consciousness static and merely sequential, like a frame-by-frame cinematic film that “creates” motion above a certain frame rate, or is it dynamic and continuously unfolding in time?

**Figure 1.**
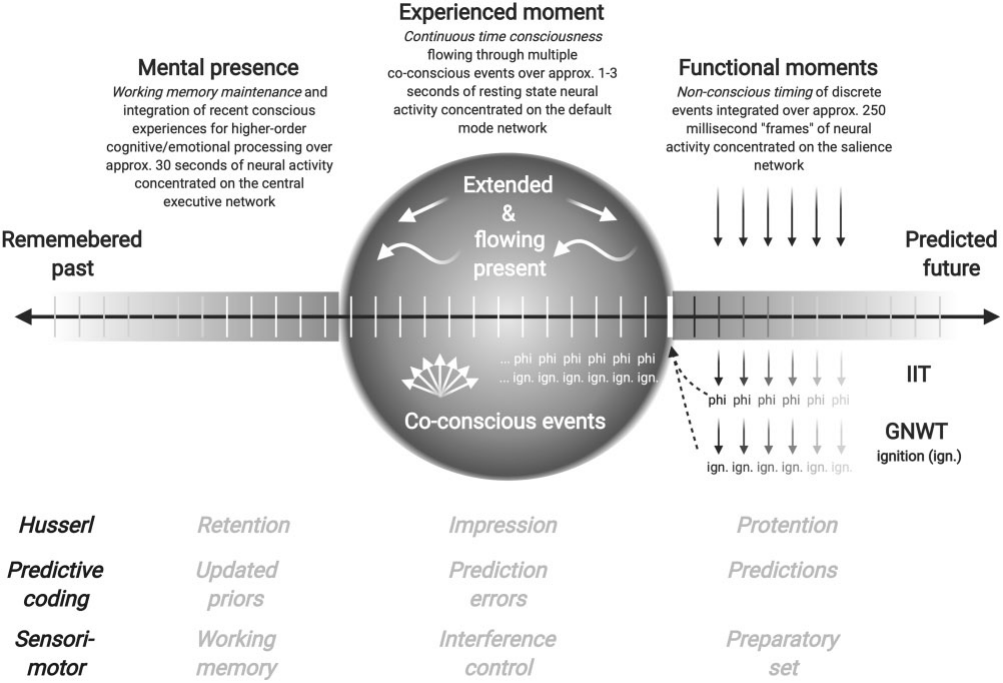
Conceptual illustration of continuous time consciousness (cf. experienced moment) that creates an extended, flowing and dynamic present integrated over 1–3 seconds discrete, unextended, and static events (cf. functional moments) integrated over approximately 250 ms. Theories of consciousness like IIT and GNWT refer primarily to only one (i.e. first or most recent) functional moment, but time consciousness incorporates multiple events that are co-conscious (i.e. experienced together but temporally ordered) and so multiple instances of phi, global ignition, or any other synchronic event. To explain time consciousness, theories of consciousness like IIT and GNWT need to extend beyond the “moment” a single stimulus enters consciousness to accord with experience that is long, continuous, and dynamic.

Phenomenological research in time consciousness holds that discrete conscious perception at shorter millisecond timescales is complemented by continuous conscious experience (cf. both perception and action) at supra-second timescales (Dorato and Wittmann, 2020), but the latter is not properly addressed in current theories of consciousness like IIT and GNWT. Figure 1 illustrates the current state of theories of theories of consciousness like IIT and GNWT in relation to time consciousness research, showing how discrete events (cf. functional moments) create a stream of consciousness that flows from right to left, and also how consciousness (cf. the experienced moment) extends across multiple events in order to create time consciousness that is long, continuous, and dynamic.

## Long, Continuous, and Dynamic: Time Consciousness Can Remedy (Literal) Shortcomings in Theories of Consciousness

Even researchers who deny (or more implicitly ignore) meaningful phenomenological analyses nevertheless still rely on subjective experience when interpreting experimental results (e.g. “seeing” a target, or not). To accommodate this epistemological fact, and in sympathy with the complementary notion regarding consciousness by Velmans (2009), we propose a triadic approach to time that simultaneously explains phenomenal, behavioral, and neural dimensionality in cognitive neuroscience, in general, and in consciousness research, in particular. Other recent proposals share this 3-fold emphasis and come to similar conclusions regarding the issues IIT faces around discrete versus continuous time (Winters 2020). A pertinent example of this type of approach is in how Schmidt *et al.* (2016) parse out the neural, phenomenological, and behavioral/psychological aspects of the Libet paradigm to conclude that slow cortical potentials represent dynamic changes in “readiness” to act and not, as some argue, a discrete “moment” of conscious decision-making.

Such a triadic approach could serve as a stage for some of the adversarial exchanges that are taking place between theories like IIT and GNWT (Reardon 2019). This is especially true given that both IIT and GNWT have thus far concentrated on functional moments. If, e.g. GNWT was able to explain what IIT could not, given that it is not prone to the same isomorphic requirements (Negro 2020; Tsuchiya *et al.* 2020), then an experimental result could clearly favor one theory over the other.

Time consciousness could thus help to resolve the phenomenological disconnect in consciousness science. The main advantage of studies into time consciousness over other methodological approaches is that, whereas qualia are not at all instantiated in neural activity (e.g. neurons need not recreate the color red in a Cartesian theatre), time is. It is therefore possible to describe, not just a neural correlate, but also a neural basis or a neural mechanism of time consciousness. This could be an important stepping-stone toward more general neural mechanisms or bases for phenomenal consciousness itself, so long as the neural activity is sufficiently extended. Time consciousness could be operationalized as an empirical subject of enquiry without falling into the trap, highlighted above, of qualia realism versus illusionism. Borrowing the language of Doerig *et al.* (2019a) above, it simply cannot be that time consciousness only “seems” to extend in time, unless one would also like to deny that time exists, which is a position that seems untenable. The experience of time is not a quale in the strict sense of the word, and experienced moments are not qualia. Time is more abstract like space, number, size, and other magnitudes that one can derive from or apply to qualia (Bueti and Walsh 2009) and so time forms part of the perceptual field within which qualia contents are experienced (Dorato and Wittmann 2020).

We have discussed the experienced moment a lot in this article but there is a longer form of phenomenal experience called “mental presence” that maintains cognitive operations and emotional feelings through working memory activity over approximately 30 s, as it is related to working-memory span (Wittmann 2011; Dorato and Wittmann 2015, 2020). “Working memory provides a temporal bridge between events—both those that are internally generated and environmentally presented—thereby conferring a sense of unity and continuity to conscious experience” (Goldman-Rakic, 1997, 559). Northoff and Huang (2017) propose that the TTC extends conscious activity over even longer timeframes, perhaps up to hours in duration, which suggests possible parallels between long-term and lifelong memory abilities and long-term and lifelong modes of experience (Kent *et al.* 2019). Whatever the upper limit, what is clear is that synchronic, discrete time represents the lower temporal limit and as such is only the entry point for time consciousness. There is much more to explore in terms of duration, dynamics, and emotional content.

## Conclusion

In their review paper on eight different theories of consciousness, Northoff and Lamme (2020) show how the TCC (Northoff 2016; Northoff and Huang 2017) is most explicit about a temporo-spatial nestedness of spontaneous brain activity spanning several time scales. Northoff makes explicit reference to phenomenological analyses of time consciousness as one starting point for his consciousness theory. Even though definite neural processing mechanisms for the experience of presence and time passage have not been conclusively identified, the search for an answer will be essential for the understanding of consciousness.

In addition to the spatiotemporal dynamics of the brain, the continuous input from the body as part of an embodied system are discussed by Northoff and Lamme (2020) as necessary prerequisites of self-consciousness. Bodily signals, visceral, and somatosensory input from the peripheral nervous system not only enact subjective feelings from the body but also underpin emotional feelings and self-consciousness (Damasio 1999). We did not delve into the embodiment issue of self-consciousness too deeply because we wanted to concentrate on the issue of extended time consciousness. Recent conceptualizations build a direct link between bodily signals, self-consciousness, and subjective time (Wittmann 2013). Craig (2009a, 2009b), e.g. suggests that the experiences of self and time are constituted through emotional and bodily processes stemming from the same neural system, the interoceptive system including the insular cortex. Successive moments of self-realization and time consciousness thus would be formed by information originating within the body. Picard and Craig (2009) proposed that the continuous bodily signals advance with a frame rate of ∼8 Hz, which amounts to temporal building blocks (functional moments) of around 125 ms. In line with our idea of an extended present moment we would argue that these individual processing units have to be furthermore integrated over time to form a present moment of conscious experience (Wittmann 2014).

There are plenty of issues to be solved in order for researchers to agree on a neural model of consciousness. Here we wanted to emphasize an often under-represented aspect in the debate: time consciousness. The notion of an extended present as prerequisite for the experience of time passage is essential for a theoretical understanding of consciousness which, in the not-too-distant future, could become a very real and practical problem given the potential for artificial consciousness. Doerig *et al.* (2019b) concede that naturally occurring unfolded systems are biologically implausible given physical constraints (e.g. size) that evolution solved by implementing recursive networks like the human brain. But such constraint may not apply to artificially unfolded systems and so the danger for a science of artificial consciousness is that the debate concerning whether or how machines could be conscious could fall on ideological lines.

There is some evidence that this ideological divide already exists. From a combined GNWT and HOT theoretical perspective, Dehaene *et al.* (2017) give a functional definition that machines would “behave as if they were conscious” (p. 7) if they were simultaneously capable of broadcasting stimuli for global availability (i.e. ignition) and self-monitoring their own mental activity (i.e. metacognition). Proponents of IIT and PCT responded that this definition of consciousness lacks phenomenological grounding (Carter *et al.* 2018) but they did so without challenging any of the substantive claims about ignition or metacognition. Instead, they left the debate with an open question: “What would constitute successful demonstration of artificial consciousness?” (Carter *et al.* 2018, 400).

We propose that any *plausible* demonstration of artificial consciousness must include an agreeable definition and operationalization of time consciousness. The key is to triangulate strict isomorphisms between the duration of presented stimuli, their neural representation, and their subjective experience. All three need to be explained by a single theory of consciousness in order to satisfy our criteria for time consciousness that is long, continuous, and dynamic. Thankfully, the potential for such triangulation is embedded within the substantive claims of Dehaene *et al.* (2017): the bottom right-hand corner of Figure 2 (p. 3) shows averaged neural activity from a masking activity in response to stimulus recognition that lasts for another 1500 ms *after* an initial spike (i.e. ignition) between 300 and 500 ms. While not at all discounting HOT, self-monitoring, or metacognitive approaches, what can this *sustained* activity tell us about the long, continuous, and dynamic conscious experience of that stimulus? What else does the individual experience during that time when, according to theories of time consciousness, the recognized object *remains* conscious? What is their emotional response to the recognized object? How does it change what they expect to see next? Answers to questions like these would paint a more extended, continuous, and dynamic picture of (time) consciousness as experienced by biological organisms such as ourselves, and could therefore provide a more plausible demonstration of artificial consciousness than any offered by current theories of consciousness.

## References

[niab011-B1] Arstila V. Temporal experiences without the specious present. Aust J Philos 2018;96:287–302.

[niab011-B2] Berkovich-Ohana A , Glicksohn J. The consciousness state space (CSS)—a unifying model for consciousness and self. Front Psychol 2014;5:341. 24808870 10.3389/fpsyg.2014.00341 PMC4010789

[niab011-B3] Bird A , Reese E. Emotional reminiscing and the development of an autobiographical self. Dev Psychol 2006;42:613–26. 16802895 10.1037/0012-1649.42.4.613

[niab011-B4] Bueti D , Walsh V. The parietal cortex and the representation of time, space, number and other magnitudes. Philos Trans R Soc B Biol Sci 2009;364:1831–40. 10.1098/rstb.2009.0028 PMC2685826 19487186

[niab011-B5] Carter O , Hohwy J, van Boxtel J et al Conscious machines: defining questions. Science 2018;359:400. 10.1126/science.aar4163 29371459

[niab011-B6] Conway MA , Singer JA, Tagini A. The self and autobiographical memory: correspondence and coherence. Soc Cogn 2004;22:491–529.

[niab011-B7] Craig AD. Emotional moments across time: a possible neural basis for time perception in the anterior insula. Philos Trans R Soc B Biol Sci2009a;364:1933–42. 10.1098/rstb.2009.0008 PMC2685814 19487195

[niab011-B8] Craig AD. How do you feel–now? The anterior insula and human awareness. Nat Rev Neurosci2009b;10:59–70. 19096369 10.1038/nrn2555

[niab011-B9] Damasio A. The Feeling of What Happens: Body and Emotion in the Making of Consciousness. San Diego: Harcourt, Inc, 1999.

[niab011-B10] Dehaene S. Temporal oscillations in human perception. Psychol Sci 1993;4:264–70.

[niab011-B11] Dehaene S , Changeux J-P. Experimental and theoretical approaches to conscious processing. Neuron 2011;70:200–27. 21521609 10.1016/j.neuron.2011.03.018

[niab011-B12] Dehaene S , Lau H, Kouider S. What is consciousness, and could machines have it? Science 2017;358:486–92. 29074769 10.1126/science.aan8871

[niab011-B13] Dennett DC. Illusionism as the obvious default theory of consciousness. J Consciousness Stud. 2016;23:65–72.

[niab011-B14] Doerig A , Scharnowski F, Herzog MH. Building perception block by block: a response to Fekete et al. Neurosci Consciousness2019a;2019:niy012. 10.1093/nc/niy012 PMC6349944 30723552

[niab011-B15] Doerig A , Schurger A, Herzog MH. Hard criteria for empirical theories of consciousness. Cogn Neurosci 2020;12: 41–62. 32663056 10.1080/17588928.2020.1772214

[niab011-B16] Doerig A , Schurger A, Hess K et al The unfolding argument: why IIT and other causal structure theories cannot explain consciousness. Consciousness Cogn2019b;72:49–59. 10.1016/j.concog.2019.04.002 31078047

[niab011-B17] Dorato M , Wittmann M. The now and the passage of time. KronoScope 2015;15:191.

[niab011-B18] Dorato M , Wittmann M. The phenomenology and cognitive neuroscience of experienced temporality. Phenomenol Cogn Sci 2020;19:747–71.

[niab011-B19] Drissi-Daoudi L , Doerig A, Herzog MH. Feature integration within discrete time windows. Nat Commun 2019;10:4901. 31653844 10.1038/s41467-019-12919-7 PMC6814726

[niab011-B20] Edelman GM. The Remembered Present. New York: Basic Books, 1991.

[niab011-B21] Elliott MA , Giersch A. What happens in a moment. Front Psychol 2016;6:1905. 26779059 10.3389/fpsyg.2015.01905 PMC4703812

[niab011-B22] Fairhall SL , Albi A, Melcher D. Temporal integration windows for naturalistic visual sequences. PLoS One 2014;9:e102248. 25010517 10.1371/journal.pone.0102248 PMC4092072

[niab011-B998] Fekete T , Van de Cruys S, Ekroll V., van Leeuwen C. In the interest of saving time: a critique of discrete perception. Neuroscience of Consciousness, 2018;2018:(1). Retrieved from 10.1093/nc/niy003. PMC6007149 30042856

[niab011-B23] Fekete T , van Leeuwen C, Edelman S. System, subsystem, hive: boundary problems in computational theories of consciousness. Front Psychol 2016;7:1041. 27512377 10.3389/fpsyg.2016.01041 PMC4961712

[niab011-B24] Fraisse P. Perception and estimation of time. Annu Rev Psychol 1984;35:1–37. 6367623 10.1146/annurev.ps.35.020184.000245

[niab011-B25] Frankish K. Illusionism as a theory of consciousness. J Consciousness Stud 2016;23:11–39.

[niab011-B26] Fuchs T. Temporality and psychopathology. Phenomenol Cogn Sci 2013;12:75–104.

[niab011-B27] Fuster JM. Cortex and Mind: Unifying Cognition. Oxford: Oxford University Press, 2003.

[niab011-B28] Ganesh N. C-wars: the unfolding argument strikes back – a reply to ‘falsification & consciousness’. arXiv preprint arXiv:2006.13664, 2020.

[niab011-B29] Goldman-Rakic P. Space and time in the mental universe. Nature 1997;386:559–60. 9121577 10.1038/386559a0

[niab011-B30] Graziano MSA , Guterstam A, Bio BJ et al Toward a standard model of consciousness: reconciling the attention schema, global workspace, higher-order thought, and illusionist theories. Cogn Neuropsychol 2020;37:155–72. 31556341 10.1080/02643294.2019.1670630

[niab011-B31] Hasson U , Yang E, Vallines I et al A hierarchy of temporal receptive windows in human cortex. J Neurosci 2008;28:2539. 18322098 10.1523/JNEUROSCI.5487-07.2008 PMC2556707

[niab011-B32] Herzog MH , Drissi-Daoudi L, Doerig A. All in good time: long-lasting postdictive effects reveal discrete perception. Trends Cogn Sci 2020;24:826–37. 32893140 10.1016/j.tics.2020.07.001

[niab011-B33] Herzog MH , Kammer T, Scharnowski F. Time slices: what is the duration of a percept? PLoS Biol 2016;14:e1002433. 27070777 10.1371/journal.pbio.1002433 PMC4829156

[niab011-B34] Humphrey N. A History of the Mind: Evolution and the Birth of Consciousness. New York: Simon & Schuster, 1992.

[niab011-B35] Husserl E. On the Phenomenology of the Consciousness of Internal Time (1893–1917) (Brough J. B., Trans. Vol. Collected Works IV). Dordrecht: Springer, 1928/1991.

[niab011-B36] James W. The Principles of Psychology. New York: Henry Holt and Company, 1890.

[niab011-B37] Kent L. Duration perception versus perception duration: a proposed model for the consciously experienced moment. Timing Time Percep 2019;7:1–14.

[niab011-B38] Kent L , Van Doorn G, Klein B. Systema temporis: a time-based dimensional framework for consciousness and cognition. Consciousness Cogn 2019;73:102766. 10.1016/j.concog.2019.102766 31254738

[niab011-B39] Kiverstein J , Arstila V. Time in mind. In: Dyke H. and Bardon A. (eds.), A Companion to the Philosophy of Time. Chichester: John Wiley & Sons, 2013, 444–69.

[niab011-B40] Kleiner J. Brain states matter. A reply to the unfolding argument. PsyArXiv Preprints, 2019. doi:10.31234/osf.io/jdcfh. 10.1016/j.concog.2020.102981 32980665

[niab011-B41] Koch C. The Quest for Consciousness: A Neurobiological Approach. Englewood, CO: Roberts & Co, 2004.

[niab011-B42] Kornmeier J , Friedel E, Hecker L et al What happens in the brain of meditators when perception changes but not the stimulus? PLoS One 2019;14:e0223843. 31647833 10.1371/journal.pone.0223843 PMC6812751

[niab011-B43] Lalanne L , van Assche M, Giersch A. When predictive mechanisms go wrong: disordered visual synchrony thresholds in Schizophrenia. Schizophr Bull 2010;38:506–13. 20876220 10.1093/schbul/sbq107 PMC3330002

[niab011-B44] Lehmann D , Strik WK, Henggeler B et al Brain electric microstates and momentary conscious mind states as building blocks of spontaneous thinking: I. Visual imagery and abstract thoughts. Int J Psychophysiol 1998;29:1–11. 9641243 10.1016/s0167-8760(97)00098-6

[niab011-B45] Lerner Y , Honey CJ, Silbert LJ et al Topographic mapping of a hierarchy of temporal receptive windows using a narrated story. J Neurosci 2011;31:2906–15. 21414912 10.1523/JNEUROSCI.3684-10.2011 PMC3089381

[niab011-B46] Lloyd D. Neural correlates of temporality: default mode variability and temporal awareness. Consciousness Cogn 2012;21:695–703. 10.1016/j.concog.2011.02.016 21420319

[niab011-B47] Lockwood M. The Labyrinth of Time: Introducing the Universe. Oxford: Oxford University Press, 2005.

[niab011-B48] Marti S , Dehaene S. Discrete and continuous mechanisms of temporal selection in rapid visual streams. Nat Commun 2017;8:1955. 29208892 10.1038/s41467-017-02079-x PMC5717232

[niab011-B999] Martin B , Franck N, Cermolacce M, Falco A, Benair A, Etienne E., Weibel S., Coull J. T., Giersch A. Fragile temporal prediction in patients with schizophrenia is related to minimal self disorders. Scientific Reports 2017;7:8278 10.1038/s41598-017-07987-y. 28811493 PMC5557764

[niab011-B49] Martin B , Wittmann M, Franck N et al Temporal structure of consciousness and minimal self in schizophrenia. Front Psychol 2014;5:1175. 25400597 10.3389/fpsyg.2014.01175 PMC4212287

[niab011-B50] Mates J , Müller U, Radil T et al Temporal integration in sensorimotor synchronization. J Cogn Neurosci 1994;6:332–40. 23961729 10.1162/jocn.1994.6.4.332

[niab011-B51] McAuley JD , Jones MR, Holub S et al The time of our lives: life span development of timing and event tracking. J Exp Psychol Gen 2006;135:348–67. 16846269 10.1037/0096-3445.135.3.348

[niab011-B52] Milz P , Faber PL, Lehmann D et al The functional significance of EEG microstates—associations with modalities of thinking. Neuroimage 2016;125:643–56. 26285079 10.1016/j.neuroimage.2015.08.023

[niab011-B53] Montemayor C , Wittmann M. The varieties of presence: hierarchical levels of temporal integration. Timing Time Percep 2014;2:325–38.

[niab011-B54] Nagel T. What is it like to be a bat? Philos Rev 1974;83:435–50.

[niab011-B55] Nakajima Y , Shimojo S, Sugita Y. On the perception of two successive sound bursts. Psychol Res 1980;41:335–44. 7394122 10.1007/BF00308878

[niab011-B56] Negro N. Phenomenology-first versus third-person approaches in the science of consciousness: the case of the integrated information theory and the unfolding argument. Phenomenol Cogn Sci 2020;19:979–96.

[niab011-B57] Northoff G. Slow cortical potentials and “inner time consciousness”—a neuro-phenomenal hypothesis about the “width of present. Int J Psychophysiol 2016;103:174–84. 25678022 10.1016/j.ijpsycho.2015.02.012

[niab011-B58] Northoff G , Huang Z. How do the brain’s time and space mediate consciousness and its different dimensions? Temporo-spatial theory of consciousness (TTC). Neurosci Biobehav Rev 2017;80:630–45. 28760626 10.1016/j.neubiorev.2017.07.013

[niab011-B59] Northoff G , Lamme V. Neural signs and mechanisms of consciousness: is there a potential convergence of theories of consciousness in sight? Neurosci Biobehav Rev 2020;118:568–87. 32783969 10.1016/j.neubiorev.2020.07.019

[niab011-B60] Pfeifer E , Wittmann M. Waiting, thinking, and feeling: variations in the perception of time during silence. Front Psychol 2020;11:602. 32300325 10.3389/fpsyg.2020.00602 PMC7142212

[niab011-B61] Picard F , Craig AD. Ecstatic epileptic seizures: a potential window on the neural basis for human self-awareness. Epilepsy Behav 2009;16:539–46. 19836310 10.1016/j.yebeh.2009.09.013

[niab011-B62] Pöppel E. A hierarchical model of human time perception. Int J Psychophysiol 1989;7:357–9.

[niab011-B63] Pöppel E. A hierarchical model of temporal perception. Trends Cogn Sci 1997;1:56–61. 21223864 10.1016/S1364-6613(97)01008-5

[niab011-B64] Pöppel E , Schill K, von Steinbüchel N. Sensory integration within temporally neutral systems states: a hypothesis. Naturwissenschaften 1990;77:89–91. 2314478 10.1007/BF01131783

[niab011-B65] Reardon S. ‘Outlandish’ competition seeks the brain’s source of consciousness. Science 2019. doi:10.1126/science.aaz8800. 10.1126/science.366.6463.293 31624192

[niab011-B66] Röhricht J , Jo H-G, Wittmann M et al Exploring the maximum duration of the contingent negative variation. Int J Psychophysiol 2018;128:52–61. 29604306 10.1016/j.ijpsycho.2018.03.020

[niab011-B67] Ruhnau E. Time-gestalt and the observer. In: Metzinger T. (ed.), Conscious Experience. Thorverton, UK: Schöningh/Imprint Academic, 1995, 165–84.

[niab011-B68] Schmidt S , Jo H-G, Wittmann M et al ‘Catching the waves’ – slow cortical potentials as moderator of voluntary action. Neurosci Biobehav Rev 2016;68:639–50. 27328786 10.1016/j.neubiorev.2016.06.023

[niab011-B69] Stern W. Über psychische Präsenzzeit. Z Psychol Physiol Sinnes 1897;8:325–49.

[niab011-B70] Tagliazucchi E , Behrens M, Laufs H. Sleep neuroimaging and models of consciousness. Front Psychol 2013;4: 256. doi:10.3389/fpsyg.2013.00256. 23717291 10.3389/fpsyg.2013.00256 PMC3651967

[niab011-B71] Teng X , Cogan GB, Poeppel D. Speech fine structure contains critical temporal cues to support speech segmentation. Neuroimage 2019;202:116152. 31484039 10.1016/j.neuroimage.2019.116152

[niab011-B72] Thönes S , Stocker K. A standard conceptual framework for the study of subjective time. Consciousness Cogn 2019;71:114–22. 10.1016/j.concog.2019.04.004 31004875

[niab011-B73] Tononi G. An information integration theory of consciousness. BMC Neurosci 2004;5:42. 15522121 10.1186/1471-2202-5-42 PMC543470

[niab011-B74] Tononi G , Boly M, Massimini M et al Integrated information theory: from consciousness to its physical substrate. Nat Rev Neurosci 2016;17:450. 27225071 10.1038/nrn.2016.44

[niab011-B75] Tononi G , Koch C. Consciousness: here, there and everywhere? Philos Trans R Soc B Biol Sci 2015;370:20140167. 10.1098/rstb.2014.0167 PMC4387509 25823865

[niab011-B76] Tschacher W , Ramseyer F, Bergomi C. The subjective present and its modulation in clinical contexts. Timing Time Percep 2013;1:239.

[niab011-B77] Tsuchiya N , Andrillon T, Haun A. A reply to “the unfolding argument”: Beyond functionalism/behaviorism and towards a science of causal structure theories of consciousness. Consciousness Cogn 2020;79:102877. 10.1016/j.concog.2020.102877 32004720

[niab011-B78] van Wassenhove V , Wittmann M, Craig A et al Psychological and neural mechanisms of subjective time dilation. Front Neurosci 2011;5:1–10. 21559346 10.3389/fnins.2011.00056 PMC3085178

[niab011-B79] VanRullen R , Koch C. Is perception discrete or continuous? Trends Cogn Sci 2003;7:207–13. 12757822 10.1016/s1364-6613(03)00095-0

[niab011-B80] Varela FJ. The specious present: a neurophenomenology of time consciousness. In: Naturalizing Phenomenology: Issues in Contemporary Phenomenology and Cognitive Science. Stanford: Stanford University Press, 1999, 266–314.

[niab011-B81] Velmans M. Understanding Consciousness. Hove: Routledge, 2009.

[niab011-B82] Vogeley K , Kupke C. Disturbances of time consciousness from a phenomenological and a neuroscientific perspective. Schizophr Bull 2006;33:157–65. 17105967 10.1093/schbul/sbl056 PMC2632289

[niab011-B83] Waade PT , Olesen CL, Ito MM et al Consciousness fluctuates with surprise: an empirical pre-study for the synthesis of the free energy principle and integrated information theory. PsyArXiv Preprints, 2020. 10.1371/journal.pcbi.1011346 PMC10619809 37862364

[niab011-B84] Wang L , Bao Y, Zhang J et al Scanning the world in three seconds: Mismatch negativity as an indicator of temporal segmentation. PsyCh J 2016;5:170–6. 27678482 10.1002/pchj.144

[niab011-B85] White PA. The three-second “subjective present”: a critical review and a new proposal. Psychol Bull 2017;143:735–56. 28368147 10.1037/bul0000104

[niab011-B86] Winters JJ. The temporally-integrated causality landscape: a theoretical framework for consciousness and meaning. Consciousness Cogn 2020;83:102976. 10.1016/j.concog.2020.102976 32590193

[niab011-B87] Wittmann M. Moments in time. Front Integr Neurosci 2011;5:1–9. 22022310 10.3389/fnint.2011.00066 PMC3196211

[niab011-B88] Wittmann M. The inner sense of time: how the brain creates a representation of duration. Nat Rev Neurosci 2013;14:217–23. 23403747 10.1038/nrn3452

[niab011-B89] Wittmann M. Embodied time: the experience of time, the body, and the self. In: Arstila V. and Lloyd D. (eds.), Subjective Time: The Philosophy, Psychology and Neuroscience of Temporality. Cambridge MA: MIT Press, 2014, 507–23.

[niab011-B90] Wittmann M. Modulations of the experience of self and time. Consciousness Cogn 2015;38:172–81. 10.1016/j.concog.2015.06.008 26121958

[niab011-B91] Wittmann M. The duration of presence. In: Mölder B., Arstila V., Øhrstrøm P. (eds.), Philosophy and Psychology of Time. Cham: Springer International Publishing, 2016, 101–13.

[niab011-B92] Zahavi D. Subjectivity and Selfhood: Investigating the First-Person Perspective. Cambridge, MA: MIT Press, 2005.

[niab011-B93] Zhang J , Huang Z, Chen Y et al Breakdown in the temporal and spatial organization of spontaneous brain activity during general anesthesia. Hum Brain Mapp 2018;39:2035–46. 29377435 10.1002/hbm.23984 PMC6866328

