# Erratum to: Time consciousness: the missing link in theories of consciousness

**DOI:** 10.1093/nc/niab015

**Published:** 2021-05-25

**Authors:** Lachlan Kent, Marc Wittmann

**Affiliations:** Centre for Youth Mental Health, The University of Melbourne, 35 Poplar Road, Parkville, Victoria 3052, Australia; Orygen, 35 Poplar Road, Parkville, Victoria 3052, Australia; Institute for Frontier Areas of Psychology and Mental Health, Wilhelmstraße 3a, Freiburg i.Br. 79098, Germany

The originally published version of this manuscript was incorrectly included in a regular issue of the journal, with the citation Volume 2021, Issue 1. The paper should have been included in a special issue, with the following citation: Volume 2021, Issue 2.

The article title should have read: ‘Time consciousness: the missing link in theories of consciousness’, with the special issue information listed above the article title.

These errors have been corrected.

